# Tenotomy in Convergent Squint

**Published:** 1897-12

**Authors:** A. Bethune Patterson

**Affiliations:** Atlanta, Ga.


### Southern Medical Record.

A MONTHLY JOURNAL OF MEDICINE AND SURGERY.

VOL. XXVII. ATLANTA, GA., DEC., 1897. No. 12

### Original Articles.

### TENOTOMY IN CONVERGENT SQUINT.

BY A. BETHUNE PATTERSON, M. D., ATLANTA, GA.

The guiding muscles of the eye-ball are the internal and external recti, the superior and inferior recti, and the obliques, and are the muscles involved in strabismus. There are several methods of treating strabismus, which are based upon theories entertained as to the cause, and which will not be taken into discussion, as the principle object is to call attention to the too frequent and unscientific operation of cutting the internal rectus muscle in convergent squint. The indications in cross-eye have always been to straighten the eye; modern research has led us a step further, and established a second indication, that of re-establishing the function of observing with the deviating eye; for binocular vision becomes suspended soon after parallelism of the visual lines are lost; coincident with the deviation, double-vision makes its appearance, and is usually of short duration, for the squinting eye soon learns to suppress its image. The fusion of the images when once suspended, is by no means easily restored; it is a tedious and painstaking task to bring about a restoration of the physiological function, and should be of grave concern to the conscientious operator. It must not be understood that the straightening, or more properly the apparent straightening, has restored to the eye its former habit of seeing, but on the other hand when fusion takes place, it is proof positive of the parallelism of the lines of vision. I hold that the treatment can only be regarded as successful when these several conditions are realized, for the eye is useless and virtually blind, so long as the images are allowed to remain suppressed. Then it should be the principal object in the treatment or management of squint to restore to the eye its physiological usefulness. This becomes exceedingly difficult and in many cases impossible after cutting the internal rectus, the eye turns a little up and remains in this position; this hyperphoria becomes a grave complication and must be overcome before binocular vision is attained. It must be said that the considering of the strabismus operation from a cosmetic standpoint only is an obsolete custom; yet there are a few old operators who continue the practice of twenty years ago. It is to be regretted that this branch of eye-surgery has been neglected by many of our distinguished operators. It is true that the simple tenotomy is the quickest and easiest way of disposing of these cases, and coupled with the fact of the improvement on the appearance of the subject that follows, is satisfactory to those who are ignorant of the true conditions. In an article published in the SOUTHERN MEDICAL RECORD six or seven years ago, I protested against the indiscriminate cutting of the internal rectus in convergent squint. The following I select from a number of cases illustrative of the evils of which this paper complains, of operating wholly from a cosmetic standpoint: Miss P., who has been under my care for several years had her internal rectus muscle cut for convergent strabismus by a distinguished surgeon. A short time after the squint appeared the double vision had subsided only a few weeks. He was ignorant of the existing hypermatropia, or far sight, which was three and a half diopters. When she come under my treatment, there was still a slight convergence with the usual turning-up (eso-hyperphoria). I succeeded in re-establishing binocular vision by doing a graduated tenotomy and correcting the hypermatropia with glasses. I feel very confident that at the time of the first operation, if this hypermatropia had been corrected, and the accommodation kept quiet for some time by the instillation of atropine, that the cutting of the muscle would not have been necessary. Very many cases of convergent strabismus in children are due to far sight, and if taken in hand early, can be cured by glasses, which correct the refractive error; then again, there are a number of others due to weak external rectus, which can be cured by developing the weak muscle. A complete severance of the muscle should only be made after a due consideration of all the conditions incident to strabismus.

